# Supplementary material for: Effect of Short-Term Dietary Intervention and Probiotic Mix Supplementation on the Gut Microbiota of Elderly Obese Women
Source: Nutrients. 2019 Dec 10;11(12):3011. doi: 10.3390/nu11123011 (PMC6950529; doi:10.3390/nu11123011)
Supplement: Supplementary file 1 [file nutrients-11-03011-s001.pdf]

Supplementary Materials

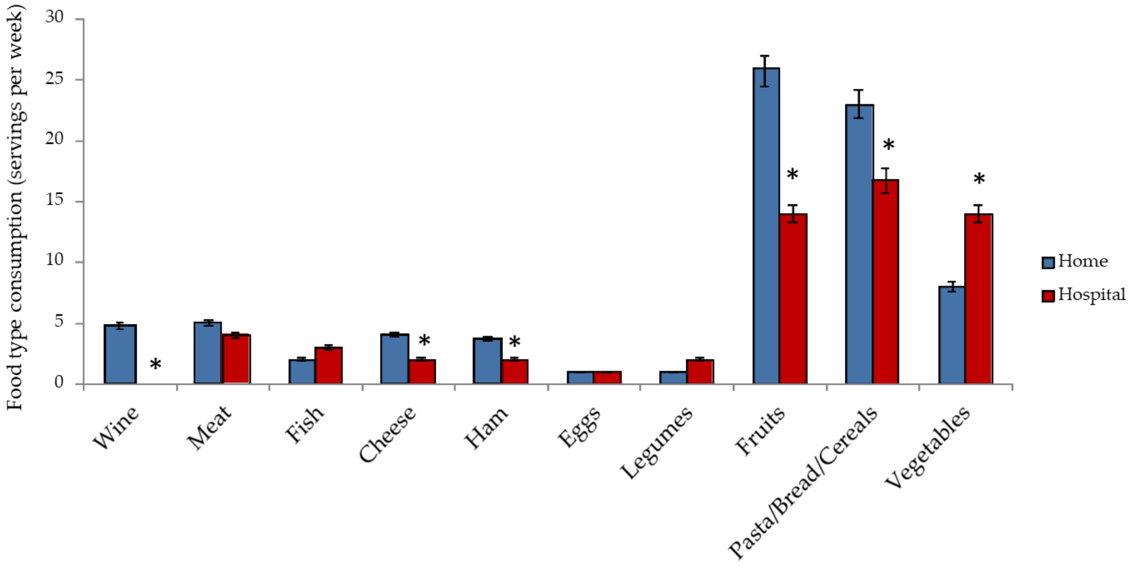

Figure S1: Mean food consumption (times a week) for the 20 elderly obese women at home (blue boxes) and during hospitalization (red boxes). There was a significant increase in vegetables and a decrease in fruit, pasta, bread, cheese, processed meat (ham) and wine consumption (*, $p < 0.05$, one-way ANOVA).

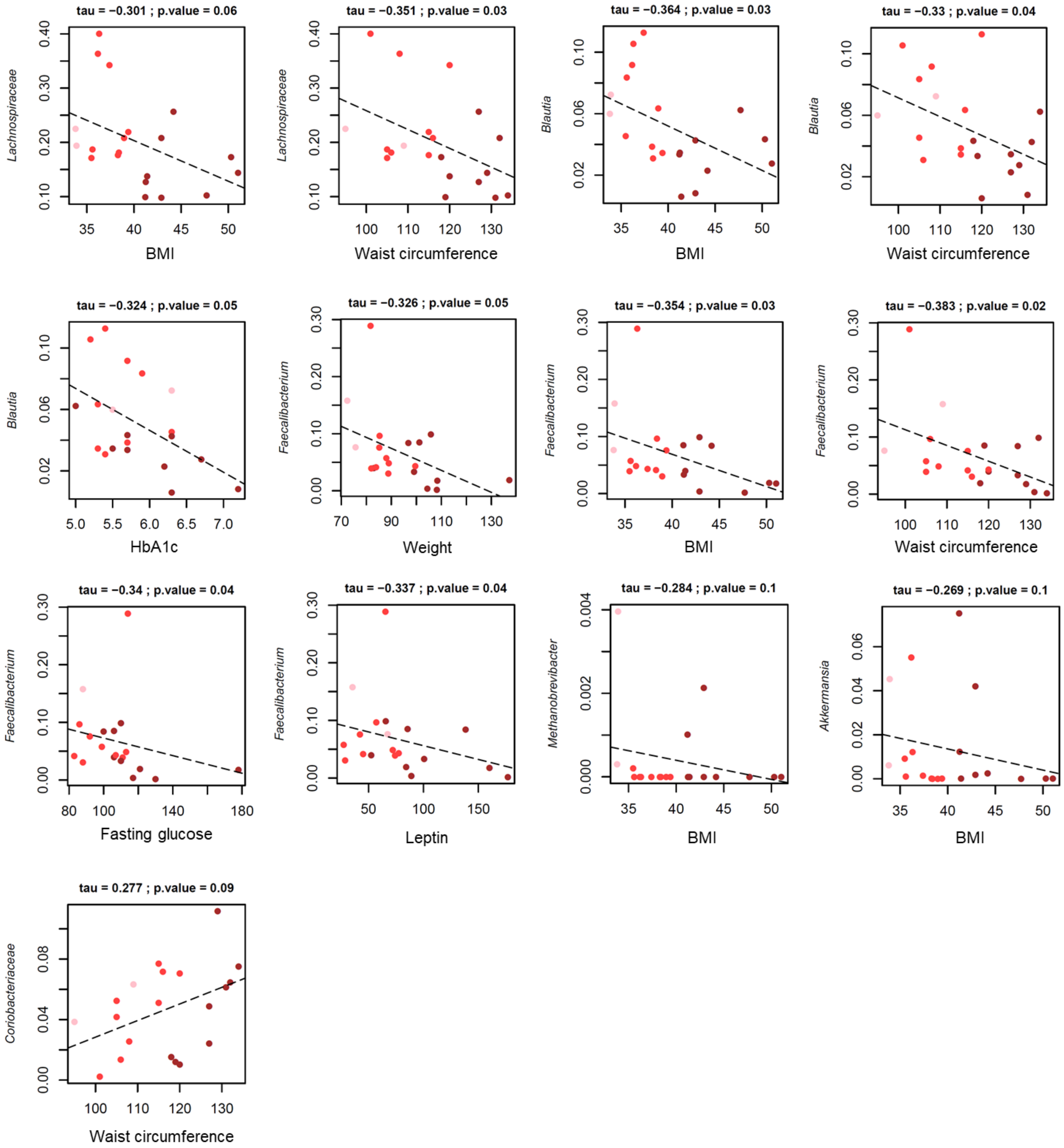

Figure S2: Scatter plots of correlations between taxon relative abundances and anthropometric/biochemical parameters in the EO cohort at the baseline. Samples from patients with class I (OB1, pink), class II (OB2, red) and class III (OB3, brown) obesity are identified with colored dots, as in Figure 1. Only statistically significant correlations ($p \leq 0.05$) or trends ($0.05 < p \leq 0.1$) based on Kendall's rank correlation test are shown.

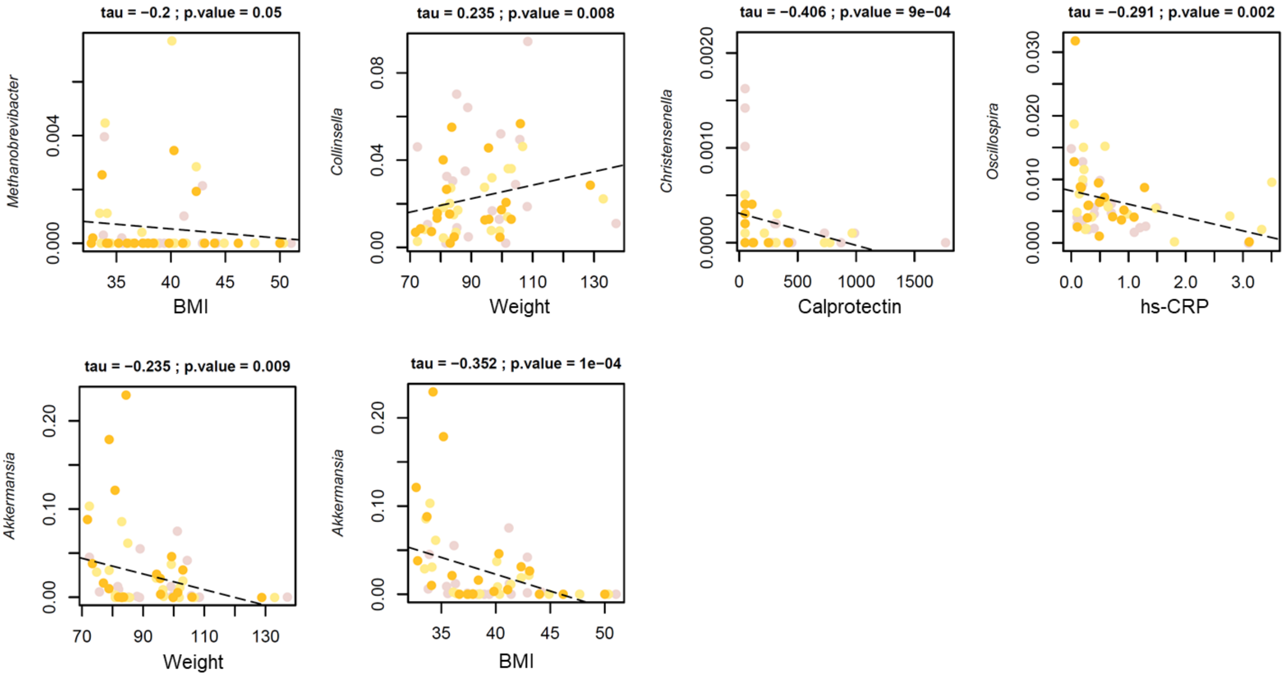

Figure S3: Scatter plots of correlations between taxon relative abundances and anthropometric/biochemical parameters in the EO cohort over the whole intervention. Samples collected at different time points are identified with colored dots, as in Figure 4 (T0, before intervention – pink; T1, after two weeks of hypocaloric balanced Mediterranean diet – light yellow; T2, after two additional weeks of diet with probiotic mixture supplementation - yellow). Only statistically significant correlations ($p \leq 0.05$) with absolute Kendall's tau ≥0.2 are shown.

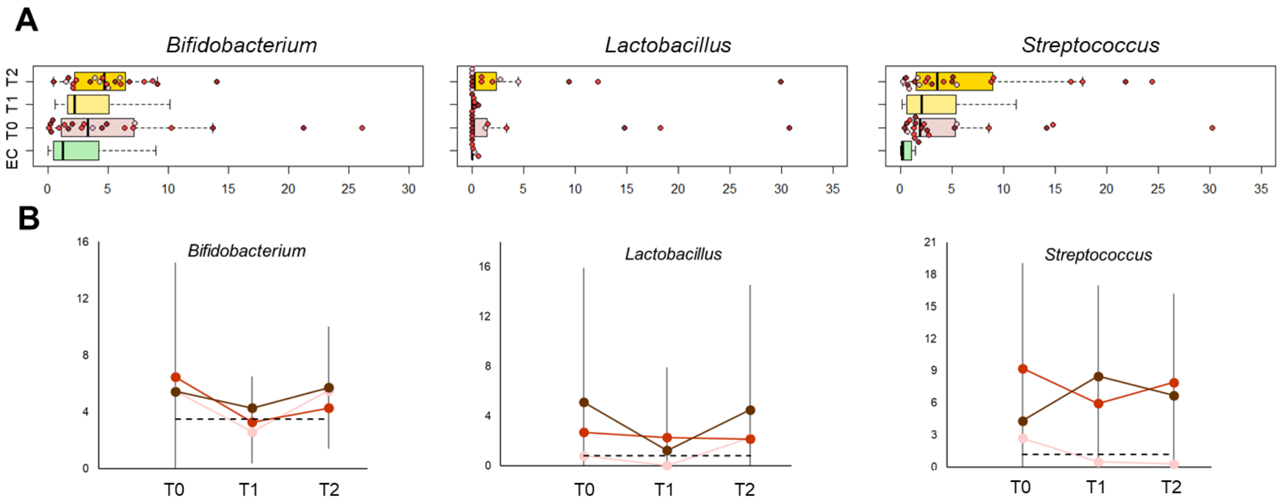

Figure S4: Relative abundance values of *Bifidobacterium*, *Lactobacillus* and *Streptococcus* over the course of the intervention. (**A**) Box plots showing the distribution of relative abundances. T0, before intervention; T1, after two weeks of hypocaloric balanced Mediterranean diet; T2, after two additional weeks of diet with probiotic mixture supplementation. Samples are identified with colored dots within boxes, based on the obesity grade as assessed at T0 and T2 (pink, class I obesity - OB1; red, class II obesity - OB2; brown, class III obesity - OB3). EC, healthy non-obese elderly. All genera increased at T2 (*Bifidobacterium*, $p = 0.02$; *Lactobacillus*, $p = 0.09$; *Streptococcus*, $p = 0.08$; Wilcoxon test). (**B**) Relative abundance dynamics for the genera of panel A. Changes in mean values (± SD) are shown by obesity grade (same color code as in A). The dotted line indicates the mean value in the control population (i.e., EC).

Table S1: Clinical and biochemical characteristics of the 20 elderly obese women over the course of the intervention. Data are shown at the baseline (T0), after two weeks of hypocaloric balanced Mediterranean diet (T1) and after two additional weeks of diet with probiotic mixture supplementation diet (T2).

**Parameter**	**T0**<br>**Mean ± SD**	**T1**<br>**Mean ± SD**	**T2**<br>**Mean ± SD**
Weight (kg)	93.8 ± 14.5	91.2 ± 13.7*	90 ± 13.5*
BMI (kg/m^2)	40.3 ± 4.9	39.3 ± 5.2	38.9 ± 6
AST (U/L)	15.9 ± 2.6	14.0 ± 3.5	15.5 ± 3.6
ALT (U/L)	14.7 ± 3.0	14.7 ± 1.0	15.2 ± 1.5
Gamma-GT (U/L)	20.4 ± 9.0	13.6 ± 4.5	17.8 ± 12.1
Total cholesterol (mg/dL)	190.9 ± 23.0	159.8 ± 31.4	170.3 ± 31.9
HDL cholesterol (mg/dL)	58.9 ± 16.3	43.2 ± 8.7	45.1 ± 10
LDL cholesterol (mg/dL)	121.5 ± 27.3	82.5 ± 25.7*	88.6 ± 28.9*
Triglycerides (mg/dL)	124.5 ± 45.5	118.3 ± 32.7	130.7 ± 37.5
hs-CRP (mg/dL)	0.7 ± 0.7	0.7 ± 0.8	1.0 ± 0.9
Fasting glucose (mg/dL)	107.6 ± 20.4	100.9 ± 13.1	103.3 ± 14.1
Insulin (μU/mL)	13.9 ± 8.6	10.3 ± 4.2	12.3 ± 4.7
Leptin (ng/mL)	77.3 ± 39.8	67.3 ± 46.1	70 ± 49.5
Adiponectin (μg/mL)	14.5 ± 8.1	15.4 ± 8.7	12.5 ± 7.3
Malondialdehyde, MDA (μmol/L)	7.3 ± 1.7	8.1 ± 1.7	8.5 ± 2.5

Data are expressed as mean ± SD. * $p < 0.05$ (one-way ANOVA for repeated measures, T1 or T2 vs. T0).
